# Inhibition of Human MCF-7 Breast Cancer Cells and HT-29 Colon Cancer Cells by Rice-Produced Recombinant Human Insulin-Like Growth Binding Protein-3 (rhIGFBP-3)

**DOI:** 10.1371/journal.pone.0077516

**Published:** 2013-10-15

**Authors:** Stanley C. K. Cheung, Xiaohang Long, Lizhong Liu, Qiaoquan Liu, Linlin Lan, Peter C. Y. Tong, Samuel S. M. Sun

**Affiliations:** 1 State Key Laboratory of Agrobiotechnology, School of Life Sciences, The Chinese University of Hong Kong, Hong Kong SAR, China; 2 Department of Medicine and Therapeutics, The Chinese University of Hong Kong, Prince of Wales Hospital, Hong Kong SAR, China; 3 Key Laboratory of Plant Functional Genomics of the Ministry of Education, Agricultural College, Yangzhou University, Jiangsu, China; TGen, United States of America

## Abstract

**Background:**

Insulin-like growth factor binding protein-3 (IGFBP-3) is a multifunctional molecule which is closely related to cell growth, apoptosis, angiogenesis, metabolism and senescence. It combines with insulin-like growth factor-I (IGF-I) to form a complex (IGF-I/IGFBP-3) that can treat growth hormone insensitivity syndrome (GHIS) and reduce insulin requirement in patients with diabetes. IGFBP-3 alone has been shown to have anti-proliferation effect on numerous cancer cells.

**Methodology/Principal Findings:**

We reported here an expression method to produce functional recombinant human IGFBP-3 (rhIGFBP-3) in transgenic rice grains. Protein sorting sequences, signal peptide and endoplasmic reticulum retention tetrapeptide (KDEL) were included in constructs for enhancing rhIGFBP-3 expression. Western blot analysis showed that only the constructs with signal peptide were successfully expressed in transgenic rice grains. Both rhIGFBP-3 proteins, with or without KDEL sorting sequence inhibited the growth of MCF-7 human breast cancer cells (65.76 ± 1.72% vs 45.00 ± 0.86%, *p* < 0.05; 50.84 ± 1.97% vs 45.00 ± 0.86%, *p* < 0.01 respectively) and HT-29 colon cancer cells (65.14 ±3.84% vs 18.01 ± 13.81%, *p* < 0.05 and 54.7 ± 9.44% vs 18.01 ± 13.81%, *p* < 0.05 respectively) when compared with wild type rice.

**Conclusion/Significance:**

These findings demonstrated the feasibility of producing biological active rhIGFBP-3 in rice using a transgenic approach, which will definitely encourage more research on the therapeutic use of hIGFBP-3 in future.

## Introduction

Insulin-like growth factor binding proteins (IGFBPs) belong to a family of cysteine-rich multifunctional proteins that bind IGF with high affinities. Of the six IGFBPs, IGFBP-3 is the most abundant IGFBP in human serum produced by hepatic Kupffer and endothelial cells [1]. From the sequence of human IGFBP-3 (hIGFBP-3) cDNA, it has been estimated that the core protein consists of 264 amino acids, and has a molecular weight of around 29 kDa [2]. However, native hIGFBP-3 is usually detected as a doublet at approximately 40- 45 kDa [3]. It has three functional domains, namely a non-conserved central domain and highly conserved cysteine-rich carboxyl- and amino-domains [4]. 

IGFBP-3 has been shown to be closely-related to growth, apoptosis, metabolism, angiogenesis and senescence acting via IGF-dependent and IGF-independent mechanisms [4-7]. As the carrier protein of IGF-I, IGFBP-3 binds to IGF-I with high affinity, limiting the bioavailability of circulating IGF-I for interactions with its receptor [8]. The complex (IGF-I/IGFBP-3) formed can be used to treat growth hormone insensitivity syndrome (GHIS) and reduce insulin requirement in patients with diabetes [4]. As for the IGF-independent actions, IGFBP-3 can induce antiproliferative and proapoptotic effects in human cancer cells. It has *in vivo* efficacy either as single agent or in chemotherapy combinations in lung cancer and colon cancer [9-11]. It is also an important mediator of human breast cancer cell [12,13]. It mediates the growth inhibitory effects of TGF-β [14,15], retinoic acid [16,17], and antiestrogens in breast cancer cells, as well as induces apoptosis by activating multiple caspases involved in a death receptor-mediated pathway, such as caspase-8 and caspase-7 [18]. Moreover, IGFBP-3 has been demonstrated to exert its tumor suppressive effects on human prostate [19] and human colorectal carcinoma [20] xenograft models. 

With the advances of plant biotechnology, plant has become an attractive bioreactor for the synthesis of recombinant proteins including industrial enzymes and pharmaceuticals at economic price [21-26]. Several plant-made pharmaceuticals are reported to be undergoing clinical trials and some of them are approaching market stage, including carrot-produced human glucocerebrosidase for Gaucher’s disease and maize-produced gastric lipase for cystic fibrosis [27]. Plant offers various advantages over traditional production systems including (1) more cost-effective and easier to scale up; (2) possessing of eukaryote post-translational modification system which synthesize recombinant proteins with correct folding, glycosylation and stability; (3) proteins made are more stable and easy to transport if produced in plant storage organ (e.g. seeds) [28].

In a proof-of-concept study, we have synthesized rhIGFBP-3 in transgenic tobacco plant previously [29]. However, it is expected its high content of nicotine and other toxic alkaloids will contribute to higher purification cost [28]. To overcome this problem, we have extended our expression technology to produce rhIGFBP-3 in transgenic rice. Apart from the production of safe and economical biopharmaceutical proteins in large quantities, rice is known to exclude any noxious chemicals as well as having low allergenicity [30]. We hypothesized that by optimizing the codons of hIGFBP-3 to plant-preferred ones, and by adding the glutelin signal peptide and the ER-retention C-terminal tetrapeptide (KDEL, Lys-Asp-Glu-Leu), functional rhIGFBP-3 could be expressed in transgenic rice grains.

## Materials and Methods

### Plasmid DNA construction

**Figure 1:**
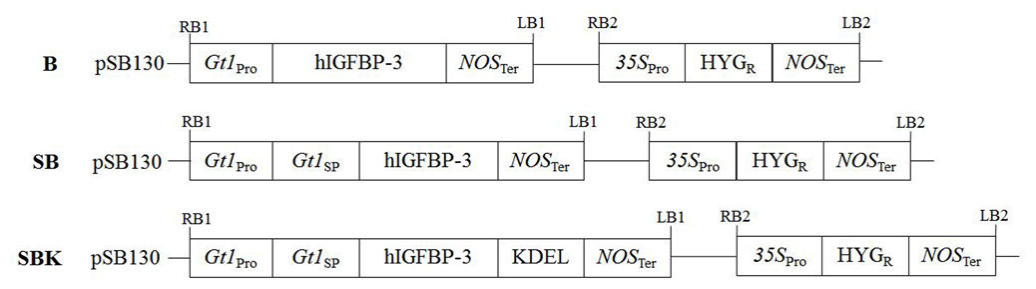
The rhIGFBP-3 expression constructs used for rice transformation. All constructs carrying the plant-codon optimized hIGFBP-3 were driven by *glutelin 1* (Gt1) promoter. The construct, pSB130/Gt1/IGFBP-3 (B), contained modified hIGFBP-3 cDNA alone (A); pSB130/Gt1/SP/IGFBP-3 (SB) contained glutelin signal peptide only (B); pSB130/Gt1/SP/IGFBP-3/KEDL (SBK) carried both glutelin signal peptide and KDEL (C). All constructs were ligated into the twin-DNA binary vector pSB130 for *Agrobacterium* transformation. The twin-DNA binary vector pSB130, contains two T-DNAs, one flanking the gene of interest driven by Gt1 promoter while the other flanking the selectable marker, hygromycin phosphotransferase (HYG). (Abbreviations: RB – right border; LB – left border).

The cDNA of *hIGFBP-3* gene was modified to plant-preferred codons as described in our previous study, with 14.8% change of codon [29]. Three constructs (Figure 1), namely promoter only construct (B), signal peptide construct (SB) and protein sorting construct (SBK) were designed and cloned into the twin T-DNA binary vector, pSB130, for *Agrobacterium*-mediated transformation [31]. The twin T-DNA binary vector contains two T-DNAs, one flanking the gene of interest driven by glutelin-1 (*Gt1*) promoter while the other flanking the selectable marker, hygromycin phosphotransferase (HYG) (Figure 1). For the construct B, the plant codon-optimized *hIGFBP-3* cDNA was amplified to introduce a 5’ BamHI and 3’ KpnI restriction sites by polymerase chain reaction (PCR) using pIGFBP-3 as template. The primers used were BBL, 5’- CGGGATCCATGGGAGCTAGCTCTGGA - 3’ and BKR, 5’- TCGTACGTCTCGTTCACTCCATGGGG - 3’. The PCR products were then ligated into pSB130/*Gt1*, resulting in pSB130/*Gt1*/hIGFBP-3 (Figure 1). The construct SB was made same as the procedure described above except for the primers used. Primers BNL, 5’- CATGCCATGGGAGCTAGCTCTGGAGGT - 3’ and BKR were used to introduce glutelin signal peptide to the gene fragment, generating pSB130/*Gt1*/SP/hIGFBP-3 (Figure 1). For the construct SBK containing KDEL, the target cDNA was PCR amplified with primers BNL and BKKR, 5’- ACGATGTCGTACGTCTCGTTCTTTCTACTCGATACTCCATGGGG - 3’ to add the KDEL protein sorting sequence in its C-terminal, forming pSB130/*Gt1*/SP/hIGFBP-3/KDEL (Figure 1). Finally, all constructs were transformed into *Agrobacterium tumefaciens* strain EHA105 by heat-shock method [31] and ready for rice transformation.

### Plant transformation and selection

Rice transformation procedure was performed in accordance with Liu et al. [32]. Briefly, mature rice seeds of an elite *japonica* rice variety Wuyunjing 9 from China were first surface sterilized in 70% ethanol for 1 minute, followed by 50% Clorox (5.25% sodium hypochlorite) and 1 drop of Tween 20 for 1 hour with shaking. After sterilization, the seeds were cultured on callus induction medium. The newly-formed callus were excised from mature embryo and immerged in the *Agrobacterium* culture harboring the expression constructs. Infected callus were placed in selection medium for the formation of resistant callus, which were then removed from the original sample and cultured in pre-regeneration and regeneration medium for shooting. The regenerated shoots were placed in rooting medium for rooting and the plantlets were finally transferred to soil and grown in greenhouse.

### Southern blot analysis

Genomic DNA of transgenic rice leave was extracted by the Cetyltrimethylammonium bromide (CTAB) method [33]. Fifteen μg of genomic DNA were digested overnight with BamHI, separated on 0.8% agarose gel and transferred to positively charged nylon membrane (Roche) using the VacuGeneXL Vacuum blotting System (Pharmacia Biotech). Hybridization and detection were carried out according to the method described in the DIG Nucleic Acid Detection Kit (Roche). Double strand DIG-labeled DNA probes (IGFBP-3) were prepared using DIG DNA labeling Kit (Roche) by PCR. The probes were heated to denature at 99°C before use.

### Northern blot analysis

Total RNA was isolated from immature rice seeds by a cold-phenol method [34] and purified by treatment with DNase I and the RNeasy Plant Mini Kit (Qiagen). Total RNA was then separated in 0.8% agarose/formaldehyde gel at 70 V for 1.5 hours and transferred to a positively charged nylon membrane (Roche) using the VacuGeneXL Vacuum blotting System (Pharmacia Biotech). Hybridization and detection were carried out according to the method described in the DIG Nucleic Acid Detection Kit (Roche). 

### Western blot analysis

Total seed protein extracts were isolated from mature dehulled seed (0.02 g) by grinding in 64 μl of protein extraction buffer (0.125 M Tris-HCl, pH 6.8). For Western blot analysis, 10 μl of total seed protein extracts (equal to 3.125 mg seed) were resolved in 10% Tricine SDS-PAGE and electrotransferred to PVDF membranes (Bio-Rad) using Towbin buffer (48 mM Tris, 39 mM Glycine and 20% methanol). Membranes were blocked with blocking solution (ICN) for 1 hour at room temperature. Goat polyclonal antibody specific for anti-human IGFBP-3 (Santa Cruz) at 1:1000 dilution and HRP-conjugated anti-goat antibody (Santa Cruz) at 1:2000 dilution were used as primary and secondary antibodies respectively. Membranes were developed using Amersham ECL Plus Western Blotting System (GE) as described in the manual.

### Anti-proliferation of MCF-7 breast cancer cells and HT-29 colon cancer cells

Total seed protein extracts were isolated from mature dehulled seed (0.08 g) by grinding in 256 μl of protein extraction buffer (0.125 M Tris-HCl, pH 6.8). The supernatants were filtered with 0.2 μm sterile filter for anti-proliferation test. MCF-7 cells were grown in Eagle’s Minimum Essential Medium (EMEM, Invitrogen) with 1.5 g/l sodium bicarbonate, 1% sodium pyruvate, 10 mg/l insulin (Sigma), 5% heat-inactivated fetal bovine serum (FBS, Hyclone), 1% penicillin-streptomycin solution and 0.1% fungizone-amphotericin solution (Sigma-Aldrich) at 37°C in a humidified atmosphere with 5% CO_2_. 0.5 × 10^3^ cells were seeded in 96-well plate with serum-containing medium for 24 hours starvation. Different concentrations of either commercial rhIGFBP-3 (Sigma) or freshly extracted rhIGFBP-3 protein were added to MCF-7 cells on every 72 hours for a total of 9 days and the cells were finally collected for MTT (3-(4,5-dimethylthiazol-2-yl)-2,5-diphenyl tetrazolium bromide) assay [35]. HT-29 cells were grown in EMEM (Invitrogen) with 2 mM Glutamine, 1% Non Essential Amino acids (NEAA) and 10% heat-inactivated FBS (Hyclone) at 37°C in a humidified atmosphere with 5% CO_2_. After 0.25% trypsin treatment, 8 × 10^3^ cells were seeded in 96-well plate with serum-containing medium for 24 hours starvation. Different concentrations of either commercial rhIGFBP-3 or total seed protein extracts from rhIGFBP-3 transgenic rice grains were added. After 48 hours incubation, 10 μl of WST-1 (Roche) solution was added to each well and incubated for 4 hours at 37°C, and the absorbance at optical wavelength 440 nm was determined with a microplate reader. Baseline was obtained by adding the protein extraction buffer (0.125 M Tris-HCl, pH 6.8) to MCF-7 and HT-29 cells.

### Glycosylation staining

Glycosylation assays were performed according to the manufacturer’s instructions (Pro-Q Emerald 300 Glycoprotein Gel and Blot Stain Kit P-21857, Invitrogen). In brief, the gel was immersed in the fixing solution (50% methanol and 10% acetic acid) and incubated at room temperature with gentle agitation for 45 min. After fixing, the gel was soaked in the washing solution (3% glacial acetic acid) for 10 min twice. It was then oxidized in the oxidizing solution (250 ml of 3% acetic acid to the bottle containing the oxidizing reagent) for 30 min, followed by washed in the washing solution for 10 min for 3 times. The Pro-Q Emerald 300 stock solution (adding 6ml *N*’*N*-Dimethylformamide (DMF) to the vial containing the Pro-Q Emerald 300 reagent) was diluted 50 folds with Pro-Q Emerald 300 staining buffer before incubating the gel in the dark for 90-120 min. The gel was incubated in the washing solution again for 10 min for 3 times. Stained gels were visualized with a UV transilluminator at 300 nm.

### Deglycosylation assay

Total seed protein extracts (3.125 mg seeds) were denatured by heating at 99°C for 10 min and then incubated with 2 mU of PNGase F or Endo H in the provided buffer (New England Biolabs) for 2 hours at 37°C. Samples were then analyzed by Western blot with commercial rhIGFBP-3 antibody in the presence of equal amount of total seed protein extracts without enzyme treatment. RNase B (10 µg) was used as the positive control to evaluate the digestion efficiency and then visualized by the glycoprotein staining kit.

### Statistical Analysis

Each experimental group consists of three biological replicates. For each biological replicate, three technical replicates were obtained. Statistical analysis was performed using a two-tailed Students *t*-test to compare each transgenic line with WT. Results with a corresponding probability value of *p* < 0.05, *p* < 0.01 and *p* < 0.001 were considered to be statistically significant, very significant and highly significant, respectively.

## Results

### Expression of rhIGFBP-3 in transgenic rice grains

**Figure 2:**
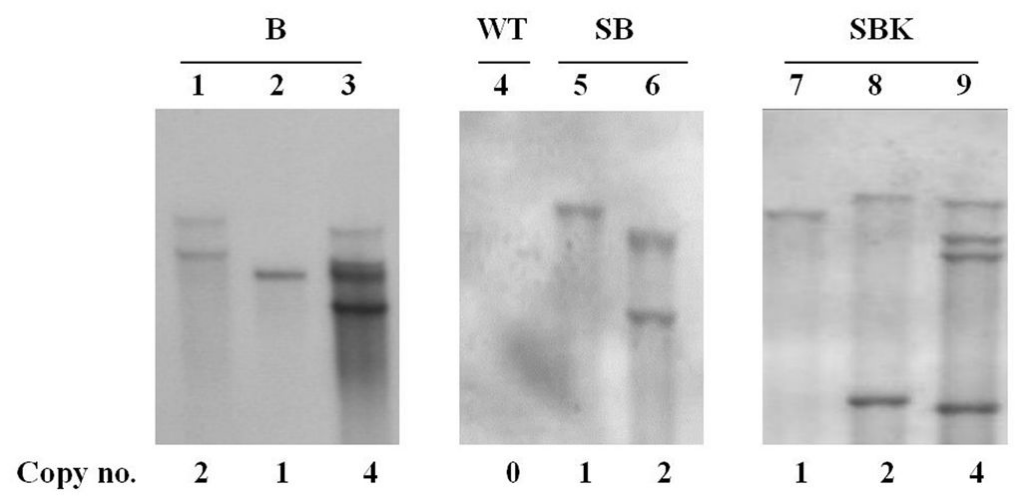
Transgenic integration of modified hIGFBP-3 in rice. Genomic DNA extracted from rice leaves of independent transformants was digested with BamHI and separated on 0.8% agarose gel, blotted on nylon membrane and hybridized with DIG-labeled hIGFBP-3 probe. Lanes 1-3: three independent pSB130/Gt1/hIGFBP-3 transformants (B1 to B3); lane 4: wild type (WT) rice plant; lanes 5-6: two independent pSB130/Gt1/SP/hIGFBP-3 transformants (SB1 to SB2); lanes 7-9: three independent pSB130/Gt1/SP/hIGFBP-3::KDEL transformants (SBK1 to SBK3).

Genomic DNA from transgenic rice leaves was isolated, digested with BamHI and used for Southern blot analysis. As the enzyme cuts only once on the expression constructs, the copy number of the transgene integration could be estimated. Most of the transformants contained 1 to 4 copies of the transgenes (Figure 2), while no signal was detected in wild type plant, suggesting that all constructs were successfully integrated into the rice genome. 

Total mRNA was extracted from developing rice seeds of independent transformants and hybridized with DIG-labeled hIGFBP-3 probe. Northern blot analysis revealed that the rhIGFBP-3 transcripts could be detected in transformants carrying the constructs, pSB130/*Gt1*/hIGFBP-3 (B), pSB130/*Gt1*/SP/hIGFBP-3 (SB), and pSB130/*Gt1*/SP/hIGFBP-3/KDEL (SBK) (Figure S1). 

**Figure 3:**
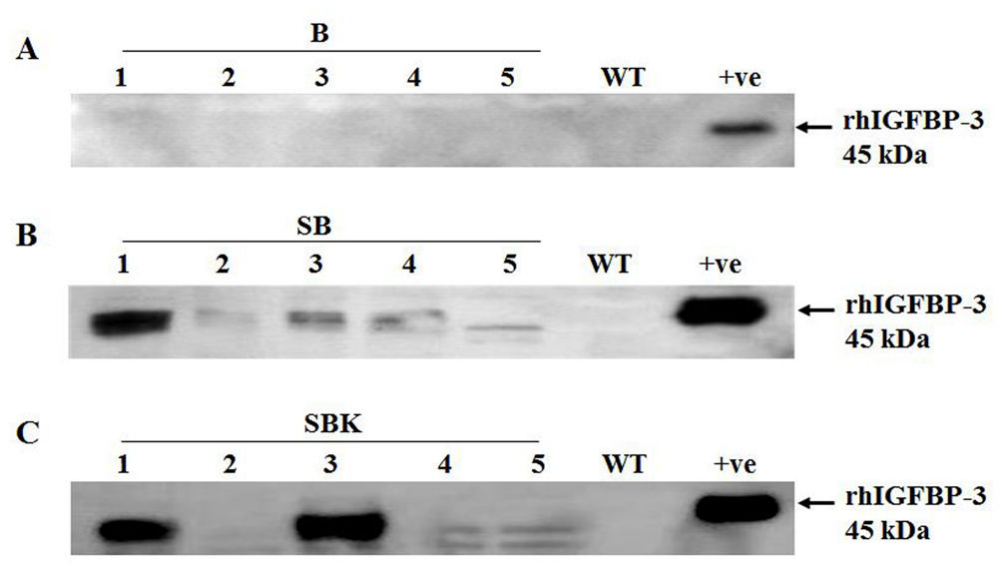
Western blot analysis of transgenic rice grains. Total protein was extracted from mature rice grains of different transformants. Recombinant hIGFBP-3 protein was used as positive control. (A) Lanes 1-5: five independent pSB130/Gt1/hIGFBP-3 transformants (B1-5); lane 6: WT; lane 7: positive control (+ve) - commercial rhIGFBP-3 protein. (B) Lanes 1-5: five independent pSB130/Gt1/SP/hIGFBP-3 transformants (SB1-5); lane 6: WT; lane 7: +ve - commercial rhIGFBP-3 protein. (C) Lanes 1-5: five independent pSB130/Gt1/SP/hIGFBP-3::KDEL transformants (SBK1-5); lane 6: WT; lane 7: +ve - commercial rhIGFBP-3 protein.

Protein extracted from transgenic rice seeds were analyzed by Western blot analysis. Commercial rhIGFBP-3 was used as positive control, with molecular weight of about 45 kDa. Signals were found in transformants harboring the constructs with glutelin signal peptide, pSB130/*Gt1*/SP/hIGFBP-3 (SB) and pSB130/*Gt1*/SP/hIGFBP-3/KDEL (SBK) only, whereas no bands were detected in transformant carrying the promoter only construct, pSB130/*Gt1*/hIGFBP-3 (B) and wild type plant. These results indicated that glutelin signal peptide enhanced the expression of rhIGFBP-3 in rice (Figure 3). Considering the expression levels of SB and SBK, line SB-57 (Figure 3B, lane 1) and line SBK-66 (Figure 3C, lane 3) showed similar expression as illustrated. Quantitation of the rice-produced rhIGFBP-3 in SB-57 and SBK-66 were further carried out by comparing with the commercial rhIGFBP-3 using Western blot analysis (Figure S2). Results showed that the expression levels of rhIGFBP-3 in SB-57 and SBK-66 were 7.5 and 6.7 µg/g seeds respectively. 

### Anti-proliferation of MCF-7 breast cancer cells and HT-29 colon cancer cells

#### Inhibitory effect of commercial rhIGFBP-3

**Figure 4:**
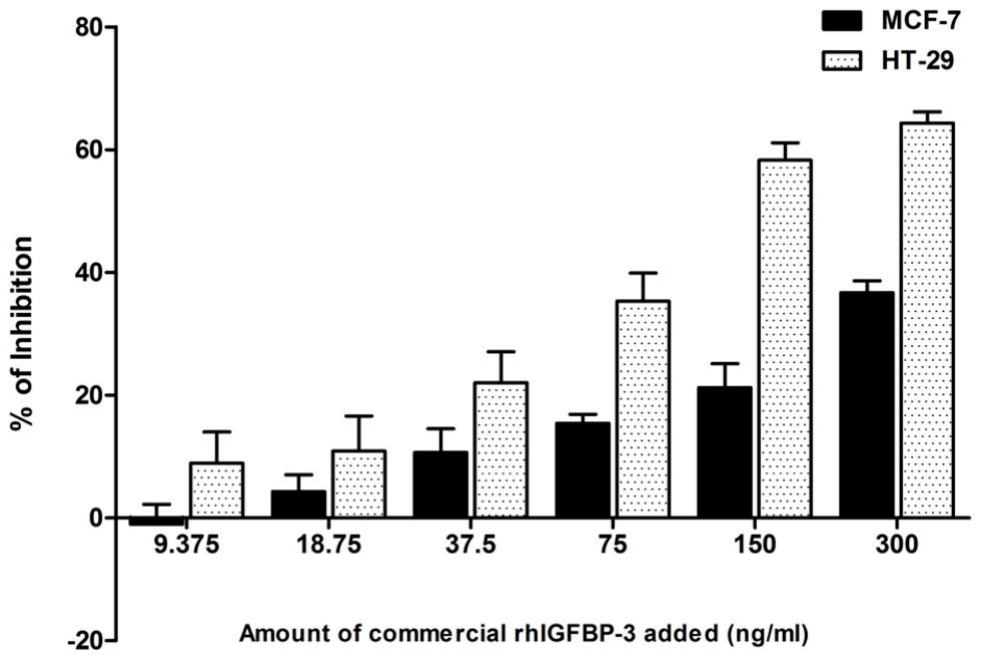
Inhibitory effect of commercial rhIGFBP-3 on human MCF-7 breast cancer cells and HT-29 colon cancer cells. Different concentrations of commercial rhIGFBP-3 ranged from 9.375 ng/ml to 300 ng/ml were used to treat MCF-7 and HT-29 cells. Data are shown as means ± SD.

Different concentrations of commercial rhIGFBP-3 were added to MCF-7 cells and HT-29 cells. Commercial rhIGFBP-3 ranging from 9.375 ng/ml to 300 ng/ml inhibited the growth of cells in a dose-dependent manner, supporting the use of these two cell models for the biological activity study of rice-produced rhIGFBP-3 (Figure 4). 

#### Inhibitory effect of rice-produced rhIGFBP-3

**Figure 5:**
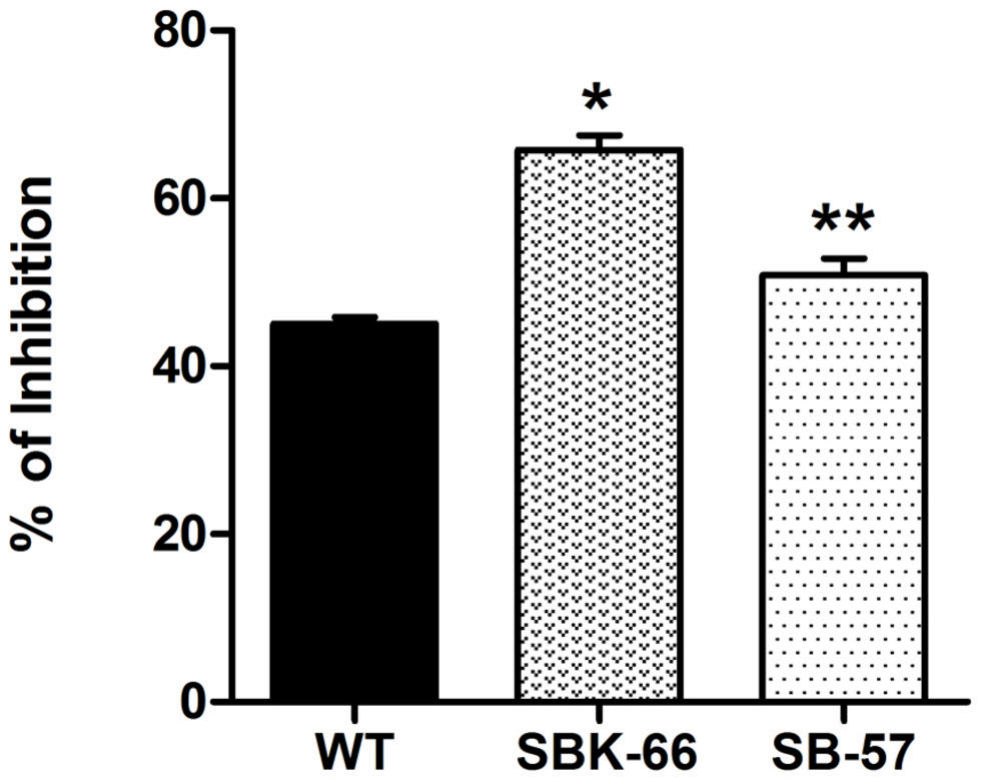
Inhibitory effect of rice-produced rhIGFBP-3 on MCF-7 human breast cancer cells. (A) Equal amount (3.125 mg seed) of WT, SB and SBK total seed protein extracts were added to the MCF-7 cells separately. Data are shown as means ± SD. *p < 0.05 and ** *p* < 0.01 denote statistically significant and very significant differences, respectively, between transgenic lines and WT.

The SBK-66 and SB-57 seed samples were selected to be analyzed in this assay as their expression levels were similar (Figure 3 and Figure S2). Equal amount (3.125 mg seed) of SBK-66, SB-57 and wild type seed total protein extracts were added to MCF-7 cells with 9 days treatment and analyzed by MTT method. The inhibitory effects of SBK-66 and SB-57 were significantly greater than that of wild type (65.76 ± 1.72% vs 45.00 ± 0.86%, *p* < 0.05; 50.84 ±1.97% vs 45.00 ± 0.86%, *p* < 0.01 respectively) (Figure 5). After deducting the inhibitory effect of wild type protein, the net inhibitory effects of SBK-66 and SB-57 were 20.76% and 5.84% respectively. When compared with the inhibitory effect of commercial rhIGFBP-3 on MCF-7 cells (Figure 4), 3.125 mg of SBK-66 and SB-57 proteins were found to possess activity equivalent to 115 ng/ml and 19 ng/ml of commercial rhIGFBP-3, respectively. 

**Figure 6:**
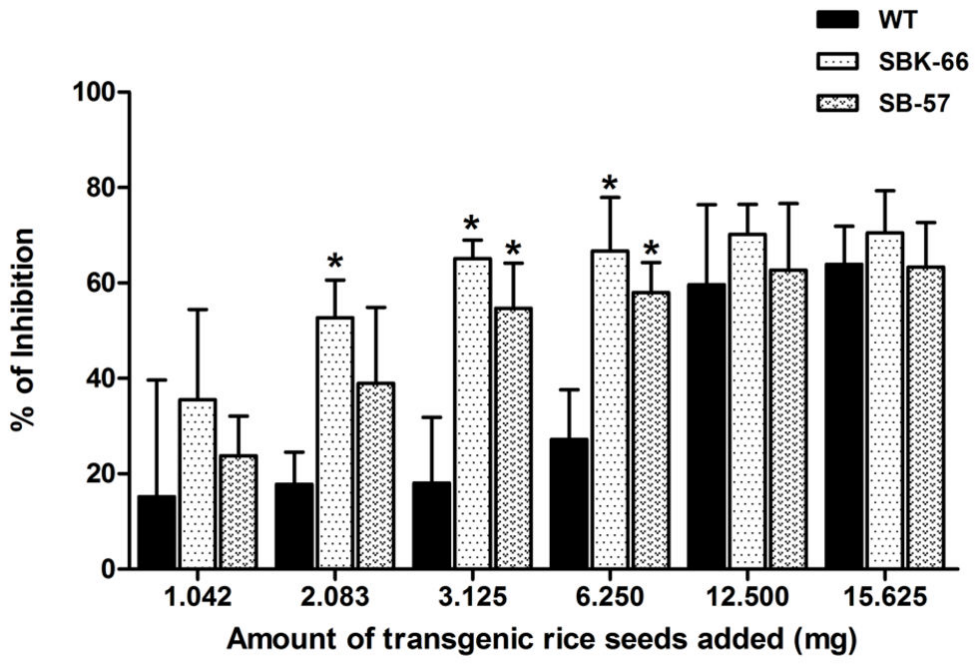
Inhibitory effect of different concentration of rice-produced rhIGFBP-3 on HT-29 human colon cancer cells. Different concentration of seed proteins (ranged from 1.042 to 15.625 mg) from WT and transgenic SBK-66 and SB-57 lines were used to treat HT-29 cells. Data are shown as means ± SD. *p < 0.05 and ** *p* < 0.01 denote statistically significant and very significant differences, respectively, between transgenic lines and WT.

The inhibitory effects of SBK and SB seeds were also studied on HT-29 cells. Different amounts of total seed protein extracts of SBK-66 and SB-57 were tested with 48 hours treatment and analyzed by WST-1 method (Figure 6). The inhibitory effects of SBK-66 and SB-57 were also found to be significantly higher than that of the wild type (65.14 ±3.84% vs 18.01 ± 13.81%, *p* < 0.05 and 54.7 ± 9.44% vs 18.01 ± 13.81%, *p* < 0.05 respectively). After deducting the inhibitory effect of wild type protein, the net inhibitory effects of SBK-66 and SB-57 were 47.13% and 36.69% respectively. When compared with the inhibitory effect of commercial rhIGFBP-3 on HT-29 cells (Figure 4), 3.125 mg of SBK-66 and SB-57 seeds were found to possess activity equivalent to 110 ng/ml and 80 ng/ml of commercial rhIGFBP-3 respectively.

#### Inhibitory effect of T2 rice-produced rhIGFBP-3

The inhibitory effect of the second generation (T2) of SBK and SB seeds was also studied on the MCF-7 and HT-29 cells. As depicted in Table 1, all lines inhibited cell growth of MCF-7 and HT-29 cells. The percentage of inhibition induced by 3.125 mg of SBK and SB T2 seeds ranged from 5 - 20% on MCF-7 cells, and 35 - 60% on HT-29 cells (after deducting the inhibition of wild type protein), which was similar to the effect of SBK and SB T1 generation.

**Table 1:** Percentage of inhibition effects of T2 rice-produced rhIGFBP-3 on MCF-7 and HT-29 cells.

**Lines**	**MCF-7**	**HT-29**
**WT**	57.133 ± 2.2611	14.600 ± 1.3200
**SBK-66**	76.670 ± 3.6482	75.256 ± 1.1757
**SB-57**	64.589 ± 4.0437	55.400 ± 4.2028

Values represent means ± SD.

### Glycosylation analysis of rice-produced rhIGFBP-3

**Figure 7:**
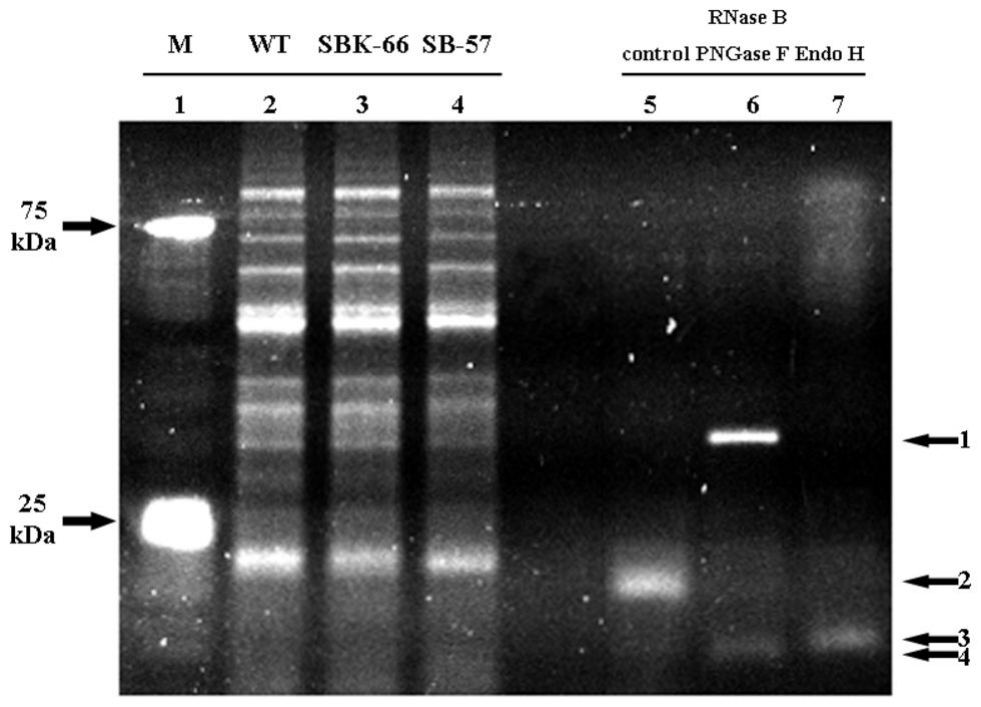
Glycoprotein staining of transgenic lines and WT line in rice seeds. Total protein was extracted from mature dehulled seeds of SB-57 and SBK-66 transgenic lines and WT. RNase B was used as the glycoprotein control. To evaluate endoglycosidase digestion efficiency, RNase B was digested by PNGase F and Endo H. Lane 1: marker; lane 2: WT; lane 3: SBK-66; lane 4: SK-57; lane 5: RNase B control without digestion; lane 6: RNase B digested by PNGase F; and lane 7: RNase B digested by Endo H. Band 1: PNGase F; band 2: RNase B; band 3: RNase B with Endo H digestion, and band 4: RNase B with PNGase F digestion.

**Figure 8:**
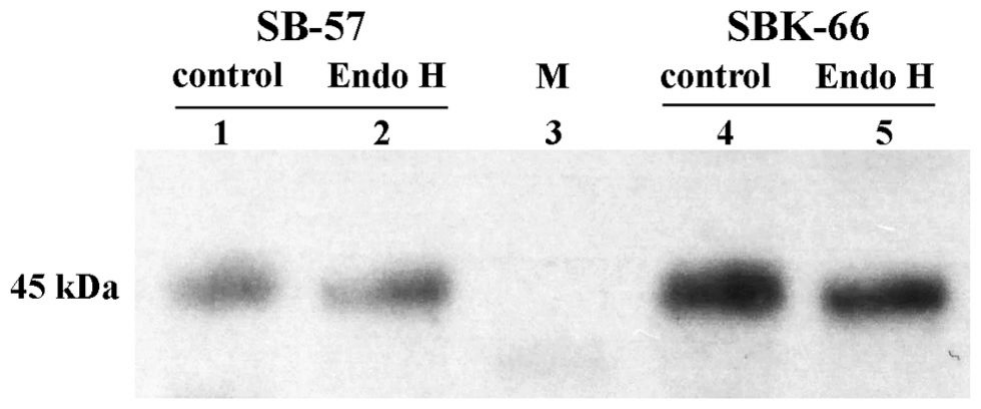
Western blot analysis of rhIGFBP-3 digested by Endo H in transgenic rice seeds. Total protein was extracted from mature dehulled seeds of SB-57 and SBK-66 transgenic lines and digested by Endo H. Lane 1: SB-57; lane 2: SB-57 digested by Endo H; lane 3: marker; lane 4: SBK-66; and lane 5: SBK-66 digested by Endo H.

To study the glycosylation of rice-produced rhIGFBP-3, glycoprotein staining was carried out. As shown in Figure 7, total seed protein extracts from SB-57, SBK-66 and WT showed similar staining patterns without apparent bands for the glycoproteins to be detected. To further characterize the glycans in the rice-produced rhIGFBP-3, we proceeded to perform endoglycosidase digestion by Endo H to evaluate the N-glycan structures of rhIGFBP-3. Results showed that there was slightly difference in protein size for both SB-57 and SBK-66 after Endo H digestion (Figure 8). This indicated that both SB-57 and SBK-66 proteins carried high-mannose-type N-glycans. The size difference between digested rhIGFBP-3 in SB-57 and SBK-66 was not obvious.

## Discussion

In this study, we demonstrated that rice grains could be used as bioreactor platform to produce functional rhIGFBP-3. It is well known that codon usage between the transgene and the expression host is essential in determining the expression of heterogeneous proteins. Although the codon usage pattern is considered to be closely related to the translational efficiency and accuracy of the gene, it does not affect the nature of the protein synthesized [36-38]. As *hIGFBP-3* cDNA is originated from human, the difference in codon usage in plants might result in poor protein expression. The *hIGFBP-3* cDNA was modified based on two seed storage proteins in plant, and was successfully expressed in transgenic tobacco seeds in our previous study [29]. 

Apart from codon usage, subcellular targeting is another factor important for higher yield of heterogeneous proteins. The compartment in which the recombinant protein accumulates strongly affects the processes of protein folding, assembly and post-translational modification. The secretory pathway is more suitable for folding and assembly than the cytosol, since it provides an oxidizing environment and an abundance of molecular chaperones but few proteases. Besides, the stability of recombinant proteins that are secreted to the apoplast is much lower than those remained in the lumen of the ER. Previous studies demonstrated that protein expression level can be greatly enhanced if the protein is retrieved to the ER lumen using KDEL [39]. Expression levels of the pea vacuole storage protein, vicilin, can be accumulated up to 100-fold in transgenic alfalfa leaves when KDEL was fused to its C-terminus [40]. In the present study, the protein expression levels of rhIGFBP-3 with (SBK) or without (SB) KDEL were similar. On the other hand, no protein was found in the transformant carrying promoter only construct (B) (Figure 3), regardless of the transcripts detected by Northern blot (Figure S1). It is expected that without glutelin signal peptide, the B protein entered the default pathway and delivered to the cytosol, where it may be susceptible to degradation by the proteolytic enzymes. In contract, the SB and SBK proteins carrying the signal peptide were supposed to enter the secretory pathway via ER [40]. After cleavage of the signal peptide in the ER, they were then transported downstream the pathway by a ‘bulk flow’ process. The SB protein, in the absence of further protein sorting sequences, was finally secreted out of the cell in the apoplast, while the SBK protein was supposed to be retained in the ER by the KDEL sequence. They were free from enzymatic degradation and hence could be stably accumulated inside the plant cell. 

When it comes to biological functions, the process of glycosylation greatly influences the physiochemical properties of recombinant proteins, including the resistance to thermal denaturation, protection form proteolytic degradation and solubility. Glycosylation takes place along the secretory pathway as proteins move from the ER through the Golgi to their final destination. Glycosidases and glycosyltransferases located in the Golgi successively modify the oligosaccharide precursor to high-mannose-type N-glycans and then into complex-type N-glycans [41]. In the central region of native IGFBP-3, three different glycosylation sites (Asn-X-Ser/Thr) are present for N-linked glycosylation [3]. It has been demonstrated that the glycans present on native IGFBP-3 are not essential for IGF binding, instead, they may affect the partitioning of IGFBP-3 between the extracellular milieu and the cell surface. Firth and Baxter [42] showed that the partially-glycosylated IGFBP-3 had more cell-surface association than the fully-glycosylated IGFBP-3 on the cell surface of the CHO transfectants, and the non-glycosylated IGFBP-3 had the greatest cell binding activity. In the present study, SBK-66 protein showed greater growth inhibition on MCF-7 (Figure 5) and HT-29 (Figure 6) cancer cells than that of the SB-57 protein. It is anticipated that SBK-66 protein, which was supposed to be retained in the ER by KDEL, contained high-mannose type N-glycans, while for SB-57 protein without KDEL, it continued its journey to the Golgi, where it was possibly heavily glycosylated with complex-type N-glycans. The complex-type N-glycans might prevent SB-57 protein from interaction with the cell surface of MCF-7 and HT-29, thus minimizing its inhibitory effect on MCF-7 and HT-29 cells. As the expression levels of SBK-66 and SB-57 were similar (Figure 3), our observations may infer that the higher anti-proliferative activity of SBK-66 on MCF-7 and HT-29 cells may be related to its lower extent of glycosylation. We have also carried out glycoprotein staining (Figure 7) to study the glycosylation of the proteins. As shown in Figure 7, total seed protein extracts from SB-57, SBK-66 and WT showed similar staining patterns without apparent bands for the glycoproteins to be detected. Hence we proceeded to perform endoglycosidase digestion to further characterize the glycans in the rice-produced rhIGFBP-3 (Figure 8). We evaluated the N-glycan structures of rhIGFBP-3 by using Endo H, which can recognize the terminal mannose residue of Mana1,3-Mana1,6-Manb1,4-GlcNAcb1,4-GlcNAc of high-mannose-type and/or hybrid-type N-glycan(s) and cleave the acetylchitobiose core of oligosaccharide [43]. Results showed that there was slightly difference in protein size for both SB-57 and SBK-66 after Endo H digestion (Figure 8). This indicated that both SB-57 and SBK-66 proteins carried high-mannose-type N-glycans, though it is hard to tell whether SBK-66 contained more high-mannose-type N-glycans [44-47] because the size difference between digested SB-57 and SBK-66 was not obvious (Figure 8). 

It is intriguing that wild type rice seed protein showed high growth inhibition on MCF-7 and HT-29 cells (Figure 5 and 6). Previous studies showed that ingestion of whole grains and grain products could reduce the risk of breast cancer [48], colon cancer [49] and prostate cancer [50]. It may be due to the rice bran, which is the brown layer of unpolished rice, contains bioactive components that possessed anti-proliferative properties [51-53], such as ferulic acid, γ-oryzanol, phytic acid, tocotrienols/tocopherols, and tricin. Studies have shown that the anticancer effects of the rice bran-derived bioactive components are mediated through their ability to induce apoptosis [54,55], inhibit cell proliferation [56], and alter cell cycle progression in malignant cells [57]. The study by Cai et al. [58] revealed the ability of tricin to arrest breast cancer cells in the G2/M phase of the cell cycle through modulating the cell cycle protein regulators, such as p34^cdc2^, Wee1 and p21^cip1^. Whole dietary rice bran contains a complex mixture of bioactive components, and each with a unique capacity to interact with multiple cellular targets to prevent the development of cancer. 

Owing to its edible nature and having low allergenicity, transgenic rice grains may be developed as seed pills for direct oral delivery [59,60]. Yang et al. [61] reported that oral administration of rice-produced anti-hypertensive peptide (RPLKPW) could reduce systolic blood pressure in rats, while Takagi et al. [62] confirmed that the rice-produced T cell epitope peptides could induce oral tolerance against pollen allergen-specific responses in transgenic mice through oral delivery. Given the problem of gastrointestinal degradation is addressed, the transgenic rhIGFBP-3 rice may also be developed as seed pills for direct oral delivery. As far as the stability of rhIGFBP-3 is concerned, SBK and SB T2 lines could inhibit cell growth of MCF-7 and HT-29 (Table 1), with 5 - 20% of inhibition on the MCF-7 cells and 35 - 60% on the HT-29 cells that were comparable to the effect of T1 generation, indicating that rhIGFBP-3 is very stable and can be retained in subsequent generations. 

There are several limitations in the present study. Firstly, the rice-derived rhIGFBP-3 was neither isolated nor purified. As illustrated in the *in vitro* study (Figure 5 and 6), total seed protein extracts of transgenic rhIGFBP-3 rice grains exerted anti-proliferative activity on MCF-7 and HT-29 cells. *In vivo* assays will be carried out before establishing the transgenic rhIGFBP-3 rice as seed pills. Secondly, only human breast cancer cells and colon cancer cells were used to test the anti-cancer properties of rice-derived rhIGFBP-3. More cancerous cell lines should be used to evaluate the anti-mitotic activity of the transgenic rhIGFBP-3 rice. Last but not least, as the extent of glycosylation on SB and SBK proteins was not clear, further characterization can be done by using mass spectrometry [43-45].

To conclude, we demonstrated the feasibility of using rice to produce functional rhIGFBP-3 using subcellular targeting approach. The anti-proliferative efficacy of transgenic rhIGFBP-3 rice grains make it possible to be developed as seed pills for direct oral delivery. In this regard, the costly purification process can be avoided. The availability of functional rhIGFBP-3 at low cost and sufficient quantity will definitely encourage more research on the therapeutic use of hIGFBP-3 in different disease states in future.

## Supporting Information

Figure S1 **Northern blot analysis of transgenic rice seeds.**
(DOC)

Figure S2 **Quantitation of rhIGFBP-3 in transgenic rice seeds by Western blot analysis.**
(DOC)
