# Supplementary material for: Inhibition of Human MCF-7 Breast Cancer Cells and HT-29 Colon Cancer Cells by Rice-Produced Recombinant Human Insulin-Like Growth Binding Protein-3 (rhIGFBP-3)
Source: PLoS One. 2013 Oct 15;8(10):e77516. doi: 10.1371/journal.pone.0077516 (PMC3797122; doi:10.1371/journal.pone.0077516)
Supplement: Figure S1 — Northern blot analysis of transgenic rice seeds. (DOC) [file pone.0077516.s001.doc]

**Supplemental Figures**


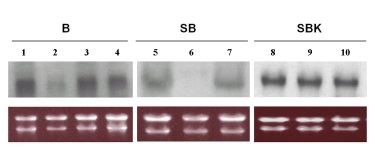


Figure S1. Northern blot analysis of transgenic rice seeds. Total mRNA extracted from developing rice seeds of independent transformants and hybridized with DIG-labeled hIGFBP-3 probe. Different independent lines were obtained for each of the three constructs and randomly selected to show the screening of the transgenic lines. Lane 1-4: four independent pSB130/Gt1/hIGFBP-3 transformants (B1-4); lane 5-7: three independent pSB130/Gt1/SP/hIGFBP-3 transformants (SB1-3); and lane 8-10: three independent pSB130/Gt1/SP/hIGFBP-3::KDEL transformants (SBK1-3).
