# Supplementary material for: Inhibition of Human MCF-7 Breast Cancer Cells and HT-29 Colon Cancer Cells by Rice-Produced Recombinant Human Insulin-Like Growth Binding Protein-3 (rhIGFBP-3)
Source: PLoS One. 2013 Oct 15;8(10):e77516. doi: 10.1371/journal.pone.0077516 (PMC3797122; doi:10.1371/journal.pone.0077516)
Supplement: Figure S2 — Quantitation of rhIGFBP-3 in transgenic rice seeds by Western blot analysis. (DOC) [file pone.0077516.s002.doc]

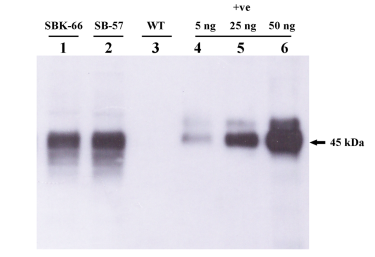


**Figure S2. Quantitation of rhIGFBP-3 in transgenic rice seeds by Western blot analysis.** Total protein (equal to 3.125 mg seeds) was extracted from mature dehulled seeds of SB-57 and SBK-66 transgenic lines and WT. Commercial hIGFBP-3 protein was used as positive control with different concentrations. Lane 1: SBK-66; lane 2: SB-57; lane 3: WT; lane 4: +ve (5 ng); lane 5, +ve (25 ng); lane 6: +ve (50 ng).
